# A Concerted Kinase Interplay Identifies PPARγ as a Molecular Target of Ghrelin Signaling in Macrophages

**DOI:** 10.1371/journal.pone.0007728

**Published:** 2009-11-04

**Authors:** Annie Demers, Véronique Caron, Amélie Rodrigue-Way, Walter Wahli, Huy Ong, André Tremblay

**Affiliations:** 1 Research Center, CHU Ste-Justine, University of Montreal, Montréal, Québec, Canada; 2 Faculty of Pharmacy, University of Montreal, Montréal, Québec, Canada; 3 Department of Biochemistry, University of Montreal, Montréal, Québec, Canada; 4 Center for Integrative Genomics, National Research Center Frontiers in Genetics, University of Lausanne, Lausanne, Switzerland; 5 Department of Obstetrics & Gynecology, University of Montreal, Montréal, Québec, Canada; Karolinska Institutet, Sweden

## Abstract

The peroxisome proliferator-activator receptor PPARγ plays an essential role in vascular biology, modulating macrophage function and atherosclerosis progression. Recently, we have described the beneficial effect of combined activation of the ghrelin/GHS-R1a receptor and the scavenger receptor CD36 to induce macrophage cholesterol release through transcriptional activation of PPARγ. Although the interplay between CD36 and PPARγ in atherogenesis is well recognized, the contribution of the ghrelin receptor to regulate PPARγ remains unknown. Here, we demonstrate that ghrelin triggers PPARγ activation through a concerted signaling cascade involving Erk1/2 and Akt kinases, resulting in enhanced expression of downstream effectors LXRα and ABC sterol transporters in human macrophages. These effects were associated with enhanced PPARγ phosphorylation independently of the inhibitory conserved serine-84. Src tyrosine kinase Fyn was identified as being recruited to GHS-R1a in response to ghrelin, but failure of activated Fyn to enhance PPARγ Ser-84 specific phosphorylation relied on the concomitant recruitment of docking protein Dok-1, which prevented optimal activation of the Erk1/2 pathway. Also, substitution of Ser-84 preserved the ghrelin-induced PPARγ activity and responsiveness to Src inhibition, supporting a mechanism independent of Ser-84 in PPARγ response to ghrelin. Consistent with this, we found that ghrelin promoted the PI3-K/Akt pathway in a Gα_q_-dependent manner, resulting in Akt recruitment to PPARγ, enhanced PPARγ phosphorylation and activation independently of Ser-84, and increased expression of LXRα and ABCA1/G1. Collectively, these results illustrate a complex interplay involving Fyn/Dok-1/Erk and Gα_q_/PI3-K/Akt pathways to transduce in a concerted manner responsiveness of PPARγ to ghrelin in macrophages.

## Introduction

Ghrelin is an acetylated 28 amino acid hormone initially identified from the stomach, which induced the release of growth hormone (GH) from the pituitary and regulates food intake, energy homeostasis and adiposity [1], [2]. Cellular signals carried by ghrelin are transduced by the growth hormone secretagogue receptor 1a (GHS-R1a), a 7-transmembrane-domain G-protein-coupled receptor mainly expressed in hypothalamus and pituitary [3]. In somatotroph cells, the activation of GHS-R1a by ghrelin induces GH release through enhanced phospholipase C activity, protein kinase C and intracellular calcium mobilization [4]. However, in concordance with the peripheral distribution of GHS-R1a, including vascular endothelium, myocardium and monocytes [5]–[7], emerging evidence indicates that ghrelin and its receptor have a variety of GH releasing-independent cardiovascular and anti-inflammatory activities [8]–[10]. Attempts to elucidate the peripheral cardiovascular effects of ghrelin have identified several signaling mechanisms involving both classical G-protein effectors and G-protein independent pathways, highlighting the complexity of GHS-R1a activation [11]–[14]. In endothelial cells, ghrelin has been shown to modulate Erk, Akt kinase, nitric oxide synthase and nuclear factor kappa B activities, in the regulation of cell proliferation and vascular inflammation [7], [12], [15]–[17]. Ghrelin also inhibited proliferation of human aortic smooth muscle cells through a cAMP/PMA activation pathway [18]. Given such complexity in GHS-R1a signaling, the molecular mechanisms underlying ghrelin downstream effects on macrophage biology have not yet been described.

Macrophages are central players for key early events in atherogenesis. The accumulation of oxidized cholesterol-rich low density lipoproteins (oxLDL) into the intima and their subsequent uptake by monocyte-derived macrophages, leads to the formation of the characteristic cholesterol-loaded foam cells. Oxidized fatty acids and oxysterols generated as a result of oxLDL uptake by macrophages, act as ligands for the nuclear receptors peroxisome proliferator-activated receptor γ (PPARγ) and liver X receptor α (LXRα) respectively, which are part of a metabolic cascade resulting in enhanced expression of downstream genes, such as apolipoprotein E and ATP-binding cassette (ABC) sterol transporters involved in cholesterol efflux [19]–[21]. An important role of PPARγ in exerting overall beneficial anti-atherosclerotic effects has been provided with the ability of thiazolidinediones, identified as high affinity synthetic PPARγ agonists with potent insulin sensitizing properties, to reduce macrophage intracellular cholesterol levels [22]–[25].

We recently reported that a growth hormone secretagogue which interacts with both the GHS-R1a receptor and the scavenger receptor CD36 markedly decreased plaque formation in apoE-null mice fed a high fat diet, a condition known to promote atherosclerosis [26], [27]. Our studies have further demonstrated that these beneficial effects were dependent on the transcriptional activation of PPARγ and enhanced expression of LXRα and ABCA1/G1 transporters, thereby leading macrophages to shunt excess cholesterol into the HDL reverse pathway [26]. The metabolic cascade involving PPARγ and LXRα was proposed as an attempt by the macrophage to enhance its ability to remove oxLDL from the vessel wall acting through the positive regulation of CD36. Although the role of CD36 receptor in mediating oxLDL uptake and PPARγ activation in macrophages is recognized, the cellular events by which GHS-R1a activation might regulate PPARγ activity and downstream gene expression remain unknown.

To understand how ghrelin and GHS-R1a might impact cholesterol metabolism in macrophages, we therefore investigated the intracellular signal transduction pathways involved in GHS-R1a activation and the ability of ghrelin to regulate PPARγ activity and dependent gene expression. Our results identify an intricate and complex kinase signaling interplay which is triggered by ghrelin and modulates in a concerted manner PPARγ-dependent transcriptional competence in macrophages.

## Results

### Ghrelin Stimulates the PPARγ-LXRα-ABC Transporters Pathway in THP-1 Macrophages

**Figure 1:**
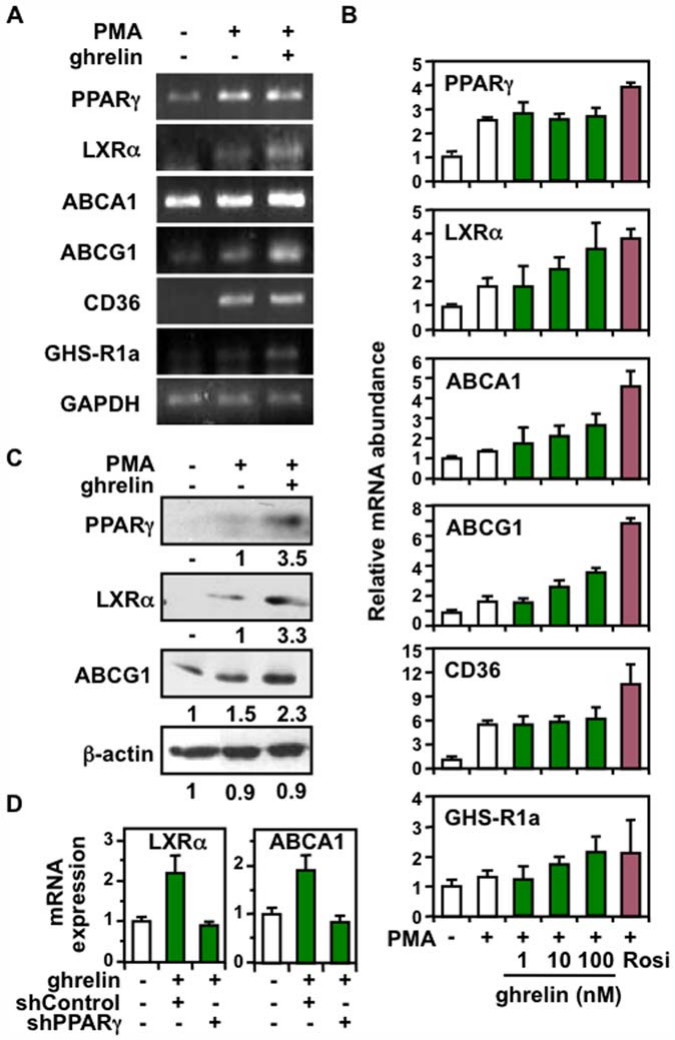
Ghrelin stimulates the PPARγ-LXRα-ABC pathway in THP-1 macrophages. (**A**) RT-PCR analysis of PMA-differentiated THP-1 macrophages treated or not with 100 nM ghrelin 48 h. Representative images are shown for the indicated genes. (**B**) Relative mRNA expression of THP-1 cells treated with increasing doses of ghrelin for 48 h. Rosiglitazone was used as a positive control of PPARγ activation. Data are presented as fold changes (±SEM) compared with undifferentiated cells, obtained from three to four separate experiments. (**C**) Representative immunoblot analysis of THP-1 cells treated with 10 nM ghrelin for 48 h. Relative fold changes are indicated. (D) PPARγ is required to mediate gene activation to ghrelin. Real-time gene expression analysis in THP-1 cells treated with 10 nM ghrelin for 48 h. PPARγ expression was silenced by infecting cells with a lentiviral-carrying shPPARγ and compared to a negative control shRNA. Results are normalized to GAPDH expression.

We previously reported that hexarelin, a GH secretagogue that interacts with both CD36 and GHS-R1a receptors, activated the PPARγ-LXRα-ABCA1/G1 transporter pathway in macrophages [26]. To evaluate the role of the GHS-R1a receptor in the regulation of cholesterol efflux effectors in the macrophage, we treated PMA-differentiated THP-1 macrophages with increasing doses of ghrelin (1, 10, and 100 nM), and measured the expression of components of the PPARγ-LXRα-ABC pathway. Our results showed that ghrelin elicited a dose-dependent increase in LXRα, ABCA1, and ABCG1 mRNA expression, as well as for GHS-R1a, with values reaching 1.8-, 1.7-, 2.2-, and 2.1-fold respectively, when treated with 100 nM ghrelin compared to untreated differentiated cells (Figs. 1A and B). These changes were paralleled with enhanced LXRα and ABCG1 protein levels (Fig. 1C). Whereas PPARγ gene expression was not significantly changed by ghrelin, a 3.5-fold increase in protein levels was observed, probably reflecting a reduced turnover of PPARγ, as also reported in THP-1 cells treated with GH secretagogues [26], [27]. CD36 mRNA and protein levels remained unchanged following ghrelin treatment, as opposed to macrophages treated with the PPARγ agonist rosiglitazone (figs. 1A and B, and data not shown). This distinct regulation of CD36 was also reported in response to hexarelin and shown to depend on a selective recruitment of PPARγ to CD36 promoter in differentiated macrophages [26], [27]. To ensure whether PPARγ was required to mediate the increase in gene expression to ghrelin, we used a shRNA lentiviral-knockdown approach to impair PPARγ expression as described [28]. We found that silencing PPARγ expression in THP-1 cells impaired the induction of LXRα and ABCA1 gene expression normally observed with ghrelin (Fig. 1D), in line with an essential role of PPARγ in mediating responsiveness to ghrelin in macrophages. These results indicate that activation of the GHS-R1a receptor by ghrelin stimulates the expression of components of the PPARγ-LXRα-ABC pathway in THP-1 macrophages.

### Ghrelin Promotes PPARγ Transcriptional Activity through the AF-1 Domain

**Figure 2:**
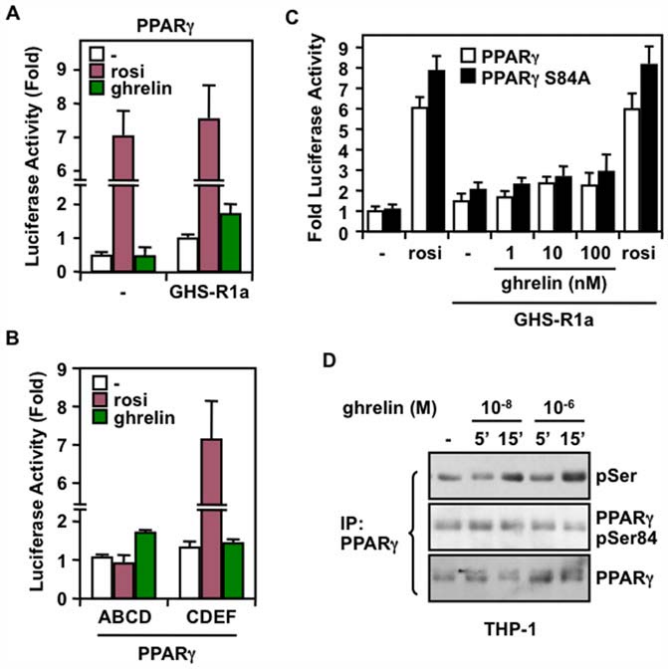
Ghrelin induces PPARγ activation and phosphorylation independently of serine-84. (**A**) Ghrelin promotes PPARγ activity in 293 cells transfected with a UAStkLuc reporter plasmid in the presence of GHS-R1a and Gal4 fusion of full-length PPARγ1 expression plasmids. Transfected cells were treated with 10 nM ghrelin or 1.4 µM rosiglitazone for 20 h and harvested for luciferase activity. Values are normalized to β-galactosidase activity and expressed as fold changes in luciferase activity compared with untreated cells expressing GHS-R1a. Error bars represent the mean ±SEM of triplicate values derived from at least three independent experiments. (**B**) Similar as in (A) except that a truncated ABCDγ construct lacking the ligand binding domain, or a CDEFγ construct lacking the N-terminal region were used. (**C**) Activation of PPARγ by ghrelin is independent of serine 84. 293 cells were transfected as in (A) with an expression vector for Gal4-PPARγ or Gal4-PPARγS84A. Cells were treated with ghrelin as indicated or with 1.4 µM rosiglitazone for 20 h. (**D**) Phosphorylation of PPARγ in serum-deprived differentiated THP-1 cells treated with increasing doses of ghrelin for the indicated time. Cell extracts were immunoprecipitated with an antibody against PPARγ and analyzed by immunoblot using antibodies against phospho-serine residue or phospho-specific to PPARγ serine-84. Samples were normalized with a different anti-PPARγ antibody.

To directly assess whether ghrelin signals to activate PPARγ, we used a luciferase reporter assay in which human embryonic kidney 293 cells were transfected in presence or absence of the GHS-R1a receptor, along with a Gal4-PPARγ expression plasmid and a UAStkLuc reporter. As shown in figure 2A, cells expressing GHS-R1a showed a 1.7-fold increase in PPARγ transcriptional activity in response to ghrelin when compared to untreated cells. To determine which part of PPARγ conferred such responsiveness to ghrelin, we used truncated ABCD and CDEF constructs of PPARγ in which the transcriptional active domains AF-2 and AF-1 were respectively removed. We found that ghrelin promoted the activity of the Gal4-ABCDγ to a similar extent as to the full-length receptor, whereas no significant activation of the CDEFγ construct was observed (Fig. 2B). These data suggest that GHS-R1a activation by ghrelin increases the ligand-independent activity of PPARγ through activation of its N-terminal AF-1 domain.

### Ghrelin Promotes PPARγ1 Phosphorylation Independent of Serine 84 in Macrophages

It has been reported that phosphorylation of AF-1 conserved serine 84 of PPARγ1 and Ser-112 of PPARγ2 by MAPK/Erk resulted in PPARγ transcriptional inhibition in adipocytes [29], [30]. Consequently, we tested the implication of this site in the modulation of PPARγ activity by ghrelin, both in transfected 293 cells and in THP-1 macrophages. As expected, mutation of Ser-84 with alanine (PPARγS84A) removed the inhibition potential of this site on PPARγ1 activation in 293 cells treated or not with rosiglitazone (Fig. 2C). Interestingly, a similar enhancement in PPARγS84A activity was observed in cells transfected with GHS-R1a and treated with ghrelin, reaching a near 3-fold increase in response to 100 nM ghrelin compared to 2.2-fold in cells transfected with wt PPARγ (Fig. 2C), indicating that PPARγS84A was responding to ghrelin, therefore precluding a role of PPARγ Ser-84 in mediating ghrelin responsiveness. To test the role of Ser-84 in the response of macrophages to ghrelin, we performed immunoblot analysis using a PPARγ phospho-Ser-84 specific antibody on cell extracts immunoprecipitated with an anti-PPARγ. No increase in phosphorylation was detected on Ser-84 site of PPARγ in response to ghrelin (Fig. 2D). However, when using a total phospho-serine antibody, we observed that ghrelin strongly stimulated in a time- and dose-dependent manner the phosphorylation of PPARγ in THP-1 cells. These results indicate that ghrelin might induce kinase signaling pathways through the GHS-R1a receptor, which promote PPARγ phosphorylation independently of Ser-84.

### The Effects of Ghrelin on PPARγ Are Independent of the Epidermal Growth Factor Receptor

**Figure 3:**
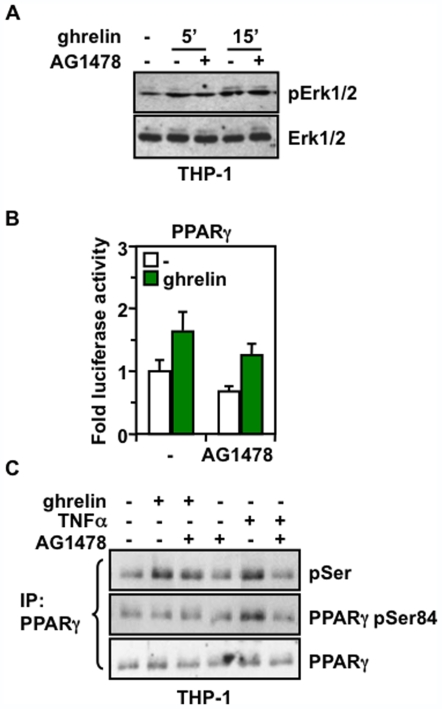
The effects of ghrelin on PPARγ are independent of the EGF receptor. (**A**) Phosphorylation of Erk1/2 in differentiated THP-1 cells serum-deprived for 16 h and treated with 5 µM of the EGFR inhibitor AG1478 for 30 min before incubation with 10 nM ghrelin for 5 and 15 min. The lysates were analyzed by immunoblot using a phospho-Erk1/2 antibody and normalized with an anti-Erk1/2 antibody. (**B**) Effect of EGFR inhibition on PPARγ activity using 293 cells transfected with UAStkLuc reporter along with Gal4-PPARγ and GHS-R1a plasmids, and then treated with 10 nM ghrelin with or without 5 µM EGFR inhibitor AG1478 for 20 h before harvested for luciferase assay. (**C**) Phosphorylation of PPARγ in THP-1 cells treated as in (A) or with 1 µM TNFα for 15 min. Cell extracts were immunoprecipitated with an antibody against PPARγ and analyzed by immunoblot with anti-phospho-serine and anti-PPARγ pSer-84 antibodies.

A wide range of extracellular signals are transduced by G-protein-coupled receptors (GPCR), and among others, it has been proposed that for several GPCR agonists, the epidermal growth factor receptor (EGFR) can be used as an intermediate to trigger MAPK/Erk activation and mitogenic signaling [31], [32]. As this role of EGFR has not been clearly defined in the peripheral actions of ghrelin, we tested whether EGFR was involved in GHS-R1a-mediated activation of MAPK by ghrelin in macrophages using the EGFR selective AG1478 inhibitor. We found that whereas a short time exposure of 5 minutes of THP-1 macrophages to ghrelin induced Erk activation in a manner partly dependent on EGFR activity, no significant effect of EGFR inhibition was observed with longer treatment periods, such as 15 and 30 minutes (Fig. 3A and data not shown), suggesting a mild and transient role of EGFR in ghrelin responsiveness of macrophages. However, whether this apparent contribution of EGFR was meaningful, it had no significant effect on PPARγ activation by ghrelin (Fig. 3B), and both total serine and Ser-84 phosphorylation of PPARγ in ghrelin-treated cells were not significantly affected upon EGFR inhibition (Fig. 3C). In contrast, the EGFR inhibitor completely abolished the increase in PPARγ phosphorylation induced by treating cells with TNF-α, a cytokine reported to promote MAPK activation and suppression of PPARγ activity in pre-adipocytes [33]. These results suggest the existence of other mechanisms independent of EGFR by which GHS-R1a regulates PPARγ activity in macrophages.

### Activation and Recruitment of Fyn Tyrosine Kinase to the GHS-R1a Receptor

**Figure 4:**
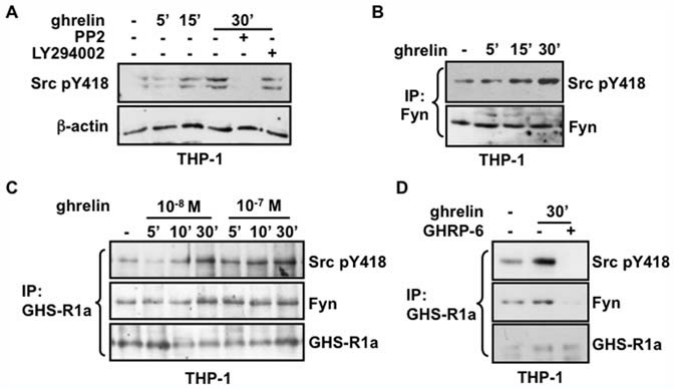
Induced recruitment and activation of Fyn kinase to the GHS-R1a receptor by ghrelin. (**A**) Ghrelin activates Src-related kinases. Differentiated THP-1 cells were serum-deprived for 16 h and incubated with 1 µM Src-related kinase inhibitor PP2 or 10 nM PI3-K inhibitor LY-294002 for 30 min before incubation with 10 nM ghrelin for the indicated times. The lysates were then analyzed by immunoblot with an anti-phospho-specific to Src Tyr-418, the activation loop site, and loading was monitored with a β-actin antibody. (**B**) Ghrelin promotes Fyn activation in THP-1 macrophages. Cells were treated as in (A) and extracts were immunoprecipitated with an antibody against Fyn, and analyzed by immunoblot using anti-Src-pY418 and anti-Fyn antibodies. (**C**) Fyn is recruited to the GHS-R1a receptor and activated by ghrelin. THP-1 cells were treated similar as in (A) and immunoprecipitation was carried out with an antibody against GHS-R1a, and analyzed by immunoblot using anti-Src-pY418 and anti-Fyn antibodies. Samples were normalized with an anti-GHS-R1a antibody. (**D**) Effect of GHS-R1a inhibition on Fyn recruitment and activation. Serum deprived THP-1 cells were pretreated with 100 nM d-Lys^3^-GHRP-6 for 30 min, followed by incubation with 10 nM ghrelin for 30 min, and then analyzed as in (C).

Non-receptor Src-family tyrosine kinases have been reported to directly interact with GPCR receptors [31], [34]. Src tyrosine kinase signaling has also been shown to play a central role in proatherogenic responses mediated by oxidized lipid metabolites in vascular smooth muscle cells implicated in atherosclerotic lesion formation [35], [36]. In an attempt to further investigate GHS-R1a signaling to PPARγ, the contribution of Src-family kinases as downstream effectors of GHS-R1a signaling was evaluated. Our findings revealed a strong time-dependent increase in response to ghrelin of Src tyrosine 418 phosphorylation (pY418), a hallmark of Src-related kinase activation, reaching nearly a 4-fold induction compared to untreated THP-1 cells (Fig. 4A). This activation was impaired in cells treated with the Src inhibitor PP2, a pyrazolopyrimidine derivative highly selective for Src, Fyn and Yes tyrosine kinases at the concentration used, whereas pretreatment with the PI3-K inhibitor LY294002 had minor effects.

In line with such activation of members of the Src family in response to ghrelin and according to the integrative role of Fyn in the metabolic regulation of lipids as recently described in peripheral tissues [37], we determined whether Fyn kinase was involved in the transduction of ghrelin signal in macrophages. Immunoprecipitation and Western analysis of activated Fyn in THP-1 cells revealed a strong time-dependent increase in Fyn tyrosine phosphorylation in response to ghrelin (Fig. 4B). To further ascertain the role of Fyn in the activation of GHS-R1a by ghrelin, we found that ghrelin induced a stronger recruitment of activated Fyn kinase to the GHS-R1a in a time- and dose-dependent manner in THP-1 cells, reaching a 5-fold increase within 30 min of treatment (Fig. 4C). Addition of the GHS-R1a antagonist (d-Lys^3^)-GHRP-6 completely abolished the recruitment of activated Fyn to the GHS-R1a receptor (Fig. 4D). These results identify Fyn kinase as a signaling intermediate in the activation of the GHS-R1a receptor.

### Fyn Kinase Restrains the Inhibitory Effect of Erk1/2 on PPARγ Activity

**Figure 5:**
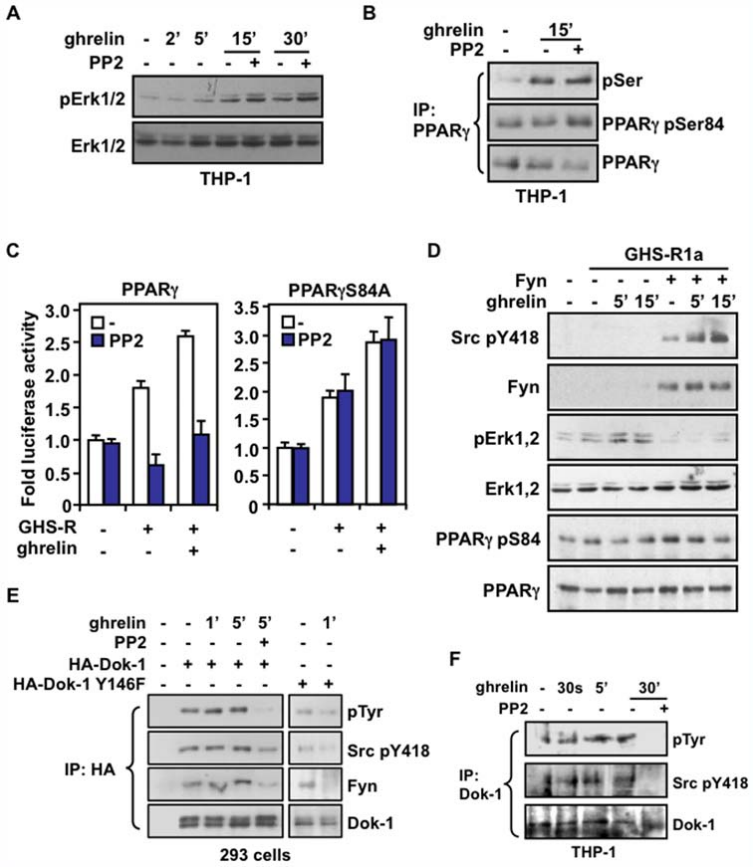
The ghrelin-induced PPARγ activation involves a negative regulation of Erk through Fyn/Dok-1 interaction. (**A**) Src inhibition increases Erk1/2 activity in differentiated THP-1 cells serum-deprived for 16 h and incubated with 1 µM PP2 for 30 min before incubation with 10 nM ghrelin for 2 to 30 min. The lysates were analyzed by immunoblot with antibodies specific to phospho-Erk1/2 and total Erk1/2. (**B**) Phosphorylation of PPARγ Ser-84 is increased by Src inhibition in THP-1 cells. Cells were treated as in (A), and lysates were immunoprecipitated with an antibody against PPARγ and analyzed by immunoblot using antibodies against phospho-serine, PPARγ pSer-84, and total PPARγ. (**C**) The PPARγ S84A mutant is insensitive to Src inhibition. 293 cells were transfected with UAStkLuc reporter in absence or presence of GHS-R1a, and with Gal4-PPARγ (*left*) or Gal4-PPARγS84A (*right*) plasmids. Cells were then treated with 10 nM ghrelin with or without 1 µM PP2 for 20 h, and harvested for luciferase assay. Normalized values are presented as fold changes ± SEM compared with untreated cells in presence of GHS-R1a. (**D**) Expression of Fyn inhibits Erk1/2 activity and PPARγ Ser-84 phosphorylation in the presence of ghrelin. 293 cells were transfected with GHS-R1a, Fyn and PPARγ expression plasmids, and then treated with 10 nM ghrelin for 5 and 15 min. Cell lysates were immunoblotted with the indicated antibodies. (**E**) Ghrelin promotes activation of Dok-1 and its recruitment to Fyn. 293 cells were transfected with Fyn and GHS-R1a expression plasmids in presence of Dok-1 or its inactive Y146F mutant, and treated with 10 nM ghrelin for the indicated time with or without 1 µM PP2. Immunoprecipitation was carried out with an antibody against HA and analyzed by immunoblot using anti-Src pTyr-418, anti-Fyn, anti-phospho-tyrosine (pTyr) and anti-Dok-1 antibodies. (**F**) Ghrelin promotes the recruitment of Dok-1 to Fyn in THP-1 macrophages. Cells were treated as in (A) and the lysates were immunoprecipitated with an antibody against Dok-1 and analyzed by immunoblot using the indicated antibodies.

Src tyrosine kinases have been involved in the ability of GPCRs to induce Erk1/2 activity [31]. Since our results suggest a role for Fyn kinase in GHS-R1a signaling, we assessed the impact of Src/Fyn inhibition on Erk1/2 activation in response to ghrelin. Interestingly, in the presence of PP2 inhibitor, THP-1 macrophages showed a respective 2- and 3-fold increase in Erk1/2 phosphorylation in response to 15 and 30 minute treatment with ghrelin, compared to cells not treated with PP2 (Fig. 5A). In addition, this activation of Erk1/2 upon Src/Fyn inhibition correlated with an increase in PPARγ phosphorylation, more specifically at Ser-84, in THP-1 cells treated with ghrelin in the presence of PP2 inhibitor (Fig. 5B). In line with the inhibitory role of Ser-84 phosphorylation on PPARγ activity, we next tested the influence of Src/Fyn inhibition on ghrelin-mediated activation of PPARγ. As shown in figure 5C, PPARγ transcriptional response to GHS-R1a expression and activation was strongly impaired in the presence of PP2 inhibitor, an effect that required the integrity of Ser-84. A similar inhibitory effect of PP2 was also observed on the activation of PPARγ to thiazolidinedione ligands (data not shown), supporting the inhibition potential of Erk on PPARγ response to ligands [38]. To ascertain the specific implication of Fyn kinase in modulating PPARγ Ser-84 phosphorylation by Erk1/2, we found that ectopic expression of Fyn in the presence of GHS-R1a in 293 cells caused a strong inhibition of Erk1/2 phosphorylation, which correlated with a time-dependent decrease of PPARγ Ser-84 phosphorylation in response to ghrelin (Fig. 5D). Altogether, our results emphasize a role for Fyn activation to restrain the inhibitory potential of Erk1/2 on PPARγ Ser-84, therefore allowing a maximal response of PPARγ to ghrelin.

### Ghrelin Promotes Activation of the Docking Protein Dok-1 and Its Recruitment to Fyn Kinase

The apparent activating role of Fyn on PPARγ activity through a negative regulation of Erk1/2 is rather intriguing and suggests that other components may modulate Src/Fyn response to ghrelin. One interesting candidate is the docking protein Dok-1 which was described as a negative regulator of the Ras/MAPK pathway and mitogenic signaling in immune cells [39], [40]. Indeed, Dok-1 becomes tyrosine phosphorylated following activation of receptor and cytoplasmic tyrosine kinases, including Src kinases Lyn and Fyn [41], and overexpression of Dok-1 was shown to inhibit Erk1/2 activation induced by Fyn in 293 cells [42]. To determine the possible implication of Dok-1 in the inhibitory effect of Fyn on Erk1/2 activity, we examined the phosphorylation status of Dok-1 and its ability to interact with Fyn kinase in response to ghrelin. Using 293 cells transfected with Dok-1 in the presence of GHS-R1a, we found that phosphorylation of Dok-1 was increased in response to ghrelin (Fig. 5E). In addition, this activated Dok-1 was co-precipitated with phosphorylated Fyn in the same conditions, indicating that ghrelin promotes activation of Dok-1 and Fyn recruitment. Both tyrosine phosphorylation of Dok-1 and recruitment of activated Fyn were completely inhibited in the presence of PP2 inhibitor and were shown to be dependent on Dok-1 Tyr-146 (Fig. 5E), a phosphorylation site involved in the ability of Dok-1 to inhibit Erk1,2 [43]. These observations on the association between Fyn and Dok-1 were also reproduced in THP-1 macrophages. Indeed, endogenous Dok-1 was shown to be increasingly tyrosine phosphorylated and recruited to activated Fyn in response to ghrelin, both of which being abolished by the PP2 inhibitor (Fig. 5F). Taken together, these findings suggest that the activation and recruitment of Fyn kinase to GHS-R1a in response to ghrelin, promotes the recruitment of Dok-1, thus forming a repressive complex by which Erk1/2 activation and its inhibitory effect on PPARγ activity through Ser-84 phosphorylation is minimized in macrophages.

### The PI3-K/Akt Pathway Promotes PPARγ Activation by Ghrelin

**Figure 6:**
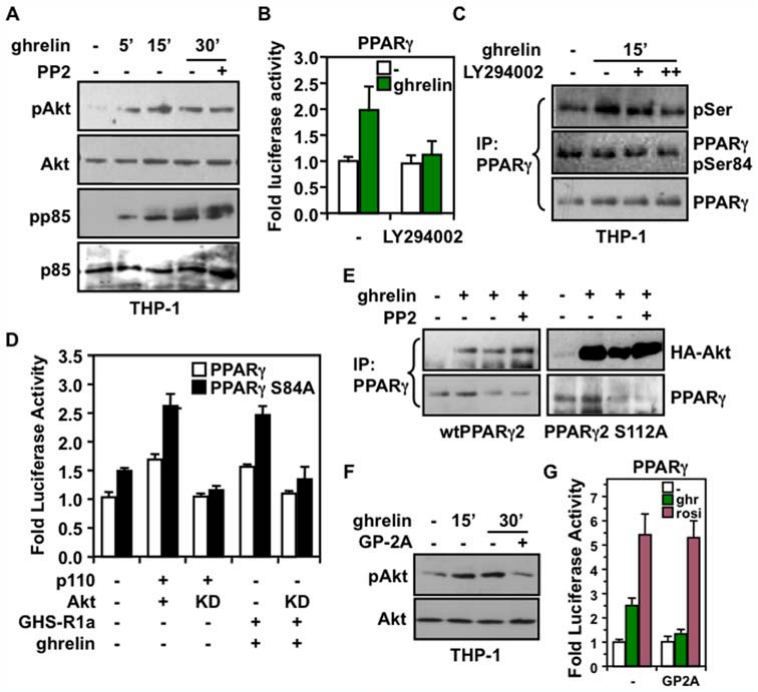
Positive regulation of PPARγ activity by ghrelin involves the PI3-K/Akt pathway in macrophages. (**A**) Ghrelin induces the PI3-K/Akt pathway in macrophages. Serum-starved THP-1 cells were treated with 10 nM ghrelin with or without 1 µM PP2 for the indicated times prior to Western analysis for phosphorylated Akt and PI3-K p85 subunit. (**B**) The PI3-K/Akt pathway is required to activate PPARγ by ghrelin. 293 cells were transfected with a PPREtkLuc reporter and expression vectors encoding PPARγ and GHS-R1a, and then treated with 10 nM ghrelin with or without 5 nM LY294002 inhibitor for 20 h. Normalized values are presented as fold changes ± SEM compared with untreated cells. (**C**) PPARγ phosphorylation in THP-1 cells treated 10 nM ghrelin in absence or presence of 5 and 10 nM LY294002. Immunoprecipitation was carried out with a PPARγ antibody and then analyzed by immunoblot using anti-phospho-serine and anti-PPARγ pSer-84 antibodies. (**D**) Akt promotes PPARγ activity independently of Ser-84. 293 cells were transfected with a UAStkLuc reporter and expression vector encoding PPARγ1 or the S84A mutant, in presence or absence of GHS-R1a. Cells were also transfected with plasmids for Akt or its kinase dead K179M (KD) mutant form in presence of constitutively active p110α subunit of PI3-K as indicated. Cells were then treated with 10 nM ghrelin or left untreated for 20 h. Normalized luciferase values are expressed as fold changes compared with untreated cells transfected with PPARγ. (**E**) Ghrelin induces recruitment of Akt to PPARγ. 293 cells were transfected with GHS-R1a and HA-Akt encoding plasmids with either PPARγ2 or PPARγ2 S112A constructs. Immunoprecipitation was carried out with a PPARγ antibody and Western blot with anti-HA and anti-PPARγ antibodies. (**F**) Protein Gα_q_ subunit is required in Akt activation by ghrelin in macrophages. THP-1 cells were treated as in (A) in presence or absence of 6 µM GP antagonist-2A. Cells were analyzed by Western blot for activated Akt. (**G**) PPARγ activation by ghrelin is dependent on Gα_q_ as determined by luciferase assay performed as in (D) in the presence or absence of 6 µM GP antagonist-2A.

Our interpretation on the inhibition potential of Fyn to restrain Erk1/2 activity through Dok-1 recruitment, therefore relieving Erk1/2 inhibition on PPARγ activity, may not fully account for PPARγ activation by ghrelin. This is especially emphasized by the fact that ghrelin could enhance Erk1/2 activity in THP-1 cells, resulting in a potential inhibition of PPARγ, and by an increased phosphorylation of PPARγ independent of Ser-84 (Fig. 5A and B). This prompted us to consider whether the PI3-K/Akt signaling pathway could be involved in the ghrelin-mediated activation of PPARγ. As such, we observed that treatment of THP-1 macrophages with ghrelin increased in a time-dependent manner the phosphorylation of Akt and of p85 regulatory subunit of PI3-K, indicating an activation of the PI3-K/Akt pathway (Fig. 6A). These responses did not seem to be mediated by Src-related kinases, as the PP2 inhibitor had no significant effect. Further supporting a role for the PI3-K/Akt pathway, we found that pretreatment with the PI3-K inhibitor LY294002, prior to stimulation with ghrelin, completely abolished the effects of ghrelin on PPARγ activity (Fig. 6B) and serine phosphorylation (Fig. 6C). Also, transient co-expression of Akt with a constitutively active p110α PI3-K in 293 cells, which results in potent activation of Akt [44], led to a 1.6- and 1.7-fold increase in the activity of PPARγ and PPARγS84A respectively, compared to mock-transfected cells (Fig. 6D), reaching values observed with ghrelin activation of GHS-R1a (Figs. 2C and 5C). This activation of PPARγ by the PI3-K/Akt pathway also correlated with an enhanced recruitment of Akt to PPARγ in response to ghrelin, as tested with the PPARγ2 isoform, which along with PPARγ1, is also expressed in macrophages (Fig. 6E). Neither PPARγ1 Ser-84, nor its conserved Ser-112 in PPARγ2, seems to behave as a critical determinant in PPARγ response to ghrelin-activated PI3-K/Akt pathway (Figs. 6C and E). Altogether, these results emphasize a predominant role of the PI3-K/Akt pathway in mediating PPARγ activation by ghrelin.

### Activation of Gα_q_ Subunit Is Required for Ghrelin-Induced Akt and PPARγ Activation

Based on recent reports that have implicated the Gq proteins in the central effects of ghrelin on GHS-R1a signaling in pituitary cells [45], [46], and on their possible convergence with the Akt pathway in endothelial cells [16], we wanted to determine whether the activation of PPARγ by ghrelin was dependent on G-protein signaling pathway in macrophages. As shown in figure 6F, pretreatment of THP-1 cells with the Gα_q_ inhibitory peptide GP-Antagonist-2A completely abrogated the activation of Akt by ghrelin. Also, Gα_q_ inhibition resulted in the suppression of the transcriptional response of PPARγ to ghrelin (Fig. 6G), whereas PKC inhibitors, such as staurosporine and GF109203X, had no significant effects (data not shown). These data identify the α subunit of Gq protein as a signaling intermediate of the GHS-R1a to activate the Akt pathway and PPARγ transcription potential in macrophages.

### The PI3-K/Akt Pathway Plays a Critical Role in the Activation of the PPARγ-LXRα-ABC Metabolic Cascade by Ghrelin

**Figure 7:**
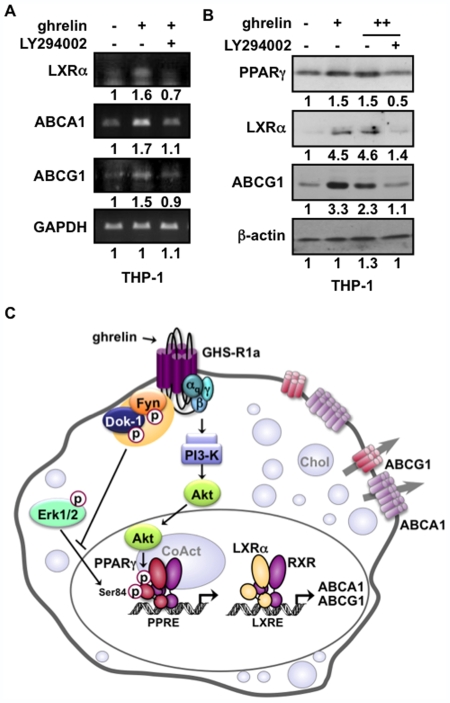
Activation of the PPARγ-LXRα-ABC transcriptional cascade by ghrelin is dependent on the PI3-K/Akt pathway. (**A**) Role of the PI3-K pathway on gene expression analysis from THP-1 cells treated with 100 nM ghrelin and 5 nM LY294002 for 48 h. Representative images and fold changes of mRNA expression are shown for the indicated genes. (**B**) Immunoblot analysis of THP-1 cells treated with ghrelin in presence or absence of 5 nM LY294002 for 48 h. Fold changes are shown relative to untreated cells. (**C**) A proposed model for the GHS-R1a-induced signaling to PPARγ in macrophages. Activation of GHS-R1a by ghrelin promotes the recruitment and activation of a Fyn/Dok-1 complex with the subsequent decrease in Erk1/2-mediated phosphorylation of PPARγ Ser-84, restraining its inhibitory potential. Ghrelin also activates the PI3-K/Akt pathway through a Gα_q_–dependent mechanism, which then promotes PPARγ AF-1 phosphorylation independently of Ser-84, resulting in receptor transcriptional activation and increase in the PPARγ-LXRα-ABCA1/G1 metabolic cascade in macrophages.

In order to establish whether activation of the PI3-K/Akt pathway was involved in the upregulation of PPARγ-dependent gene expression, we tested the effects of the PI3-K inhibitor LY294002 on the expression profile of downstream effectors, such as LXRα and ABC sterol transporters in THP-1 macrophages. Our results indicate that the increased levels observed in LXRα, ABCA1 and ABCG1 expression upon treating cells with ghrelin were strongly impaired with the inhibition of PI3-K (Fig. 7A). Similarly, these effects were paralleled with decreases in PPARγ, LXRα, and ABCG1 protein levels (Fig. 7B). These findings suggest a prominent role of the PI3-K/Akt pathway in mediating ghrelin responsiveness of macrophages to activate the PPARγ-LXRα-ABC metabolic cascade.

## Discussion

PPARγ plays a central role in coordinating the macrophage response to lipid loading through a transcriptional cascade involving LXRα and the ABC transporters A1 and G1 [19]–[21]. As such, PPARγ agonists of the thiazolidinedione family were shown to exert beneficial anti-atherosclerotic actions by promoting activation of the PPARγ-LXRα-ABC pathway and cholesterol efflux [22]–[25]. Consequently, modulation of PPARγ activity is critical to the control of cholesterol metabolism in macrophages and atherosclerotic vascular lesion progression. Emerging evidence suggests that ghrelin has important and beneficial effects on the vascular system, by ameliorating endothelial dysfunction [16], [47] and inhibiting proliferation of aortic smooth muscle cells [18]. However, ghrelin's action on the macrophage is still not well defined. In line with our previous work, which described a role for scavenger CD36 and GHS-R1a/ghrelin receptors to regulate cholesterol efflux in macrophages through a PPARγ-dependent pathway [26], [27], this study identifies a complex signaling pathway that, upon activation of GHS-R1a receptor with ghrelin, results in the enhancement of PPARγ transcriptional activity and subsequent target gene activation in THP-1 macrophages. Such modulation of PPARγ activity implicates modification of its phosphorylation status through the interplay of two signaling pathways, one being mediated by Fyn/Dok-1/Erk1/2, which mainly restrains the inhibition potential of PPARγ serine 84/112, whereas the second involves Gα_q_/PI3-K/Akt that promotes PPARγ activation. Therefore, our findings identify ghrelin as a novel regulator of PPARγ activation via GHS-R1a-mediated concerted pathways in macrophages.

Members of the MAP kinase family have been implicated in the regulation of PPARγ phosphorylation and activity in response to external signals, such as platelet derived growth factor (PDGF), epidermal growth factor (EGF), and insulin [29], [48], [49]. In particular, phosphorylation of PPARγ1/2 at conserved MAPK consensus position 84/112 is well known to reduce the transcriptional response of PPARγ to activating ligands and mitogens in adipocytes, leading to inhibition of cell differentiation and adipogenesis [29], [30], [48]. Genetic studies are also consistent with a role of PPARγ Ser-112 in fat metabolism and glucose homeostasis, as S112A knock-in mice exhibit increased PPARγ activity with protective effects from obesity-associated insulin resistance [50]. The present study demonstrates that the positive effect of ghrelin on PPARγ transcriptional activation is associated with an increase in PPARγ phosphorylation, which is not dependent on Ser-84, even if ghrelin promoted the activation of Erk. In trying to understand the molecular basis for such ligand-independent activation of PPARγ, we found that GHS-R1a efficiently recruited and activated the Src kinase Fyn in THP-1 macrophages treated with ghrelin. This specific recruitment and activation of Fyn through GHS-R1a restrained Erk1/2 activation and PPARγ phosphorylation on Ser-84, resulting in the release of Ser-84 inhibitory potential. Recent evidence has provided a role for Fyn as an essential regulator of adipogenesis and fat cell function. The requirement of Fyn and other Src-family tyrosine kinases has been demonstrated in insulin-induced fat accumulation using *Src*, *Yes* and *Fyn* triple knock out embryonic fibroblasts stably expressing PPARγ2 [51]. Likewise, genetic disruption of *Fyn* has recently been associated with peripheral tissue insulin sensitivity through modulation of AMP kinase activity [37]. In line with such potential role of Fyn to regulate adipogenesis in a context in which PPARγ is established as essential, we identify Fyn as a metabolic regulator that modulates PPARγ transcriptional potential and downstream events in macrophages.

The Src-family tyrosine kinases are typically recognized to activate Erk1/2 in various cells [14], [31], and to our surprise, inhibiting Src kinases with PP2 enhanced the activation of Erk1/2 by ghrelin in macrophages, resulting in increased phosphorylation on PPARγ Ser-84 and consequently decreased PPARγ activity. To further characterize the molecular basis of such inhibition of Erk by Fyn in the context of ghrelin, we identified Dok-1 as a potent recruiting partner of activated Fyn kinase in response to ghrelin. Dok-1 has been reported to negatively regulate Erk1/2 for tyrosine kinase receptors such as the insulin receptor, by associating with Src [40]. We extend these findings to Fyn kinase, which by recruiting Dok-1, defines a GHS-R1a/Fyn/Dok-1 signaling pathway in macrophages, which then prevents excessive Erk1/2 activation in the presence of ghrelin. We propose that this GHS-R1a/Fyn/Dok-1 pathway is used to alleviate the inhibitory potential of Ser-84/112 phosphorylation on PPARγ activity, therefore resulting in enhanced PPARγ-dependent response to ghrelin. Consistent with such repressive role of Dok-1 on macrophage PPARγ activity, it was recently reported that Dok-1 is essential to mediate adipocyte response to insulin, by antagonizing activation of the Ras/Erk1/2 pathway that would otherwise maintain basal phosphorylation of PPARγ Ser-112 and limit its ability to promote terminal differentiation of adipocytes [52].

Our findings that Dok-1 is used to restrain the inhibitory potential of Erk on PPARγ might not fully explain how ghrelin achieves PPARγ activation in macrophages. Signaling of ghrelin receptor is complex and multiple pathways with frequent cross-talk of signaling components have been described in several cell types. Although not clearly defined for GHS-R1a receptor, the EGFR was proposed as an intermediate partner in transducing Erk activation by GPCRs [31]. Also, ghrelin was described to activate cAMP-dependent protein kinase in aortic endothelial cells [15] and several isoforms of calcium-dependent protein kinase [14], [32]. Notwithstanding their potential role in transducing ghrelin responsiveness to other cellular factors, our results indicate that these pathways are unlikely to be involved in GHS-R1a mediated PPARγ activation in macrophages.

Interestingly, the observation that ghrelin promoted activation of the PI3-K/Akt pathway, which then resulted in enhanced activation of PPARγ, provides a mean by which ghrelin can initiate positive signals to PPARγ in macrophages. Akt was described to mediate ghrelin responsiveness in various cells [11], [16], [53], [54], and this activation was recently shown to be dependent on both a Gα_i/o_-protein-coupled and β-arrestin scaffolded complex in 3T3-L1 cells [54]. In addition, expression of an activated form of Akt was described to trigger spontaneous adipocyte differentiation of 3T3-L1 preadipose cells [55], [56], in support of a role of Akt in regulating PPARγ in fat cells. Here, we demonstrate that the PI3-K/Akt pathway acts as a positive regulator of PPARγ function in macrophages, by promoting the induction of the PPARγ-LXRα-ABC metabolic cascade that governs cholesterol removal from these cells. The activation of Akt by ghrelin is a result of a distinct signaling pathway, which is independent of Src-related activity but required Gα_q_ coupling to GHS-R1a receptor, emphasizing the complexity of ghrelin signaling in macrophages.

Although PPARγ was shown to be phosphorylated independently of Ser-84 upon activation of Akt, no consensus site for Akt has be identified in PPARγ1/2, suggesting that the activation potential of Akt on PPARγ is probably indirect. Numerous transcriptional coactivators that facilitate PPARγ activity have been described, some of which being directly regulated by Akt [57]. For instance, we showed that PPARγ can benefit from the enhanced transcriptional potential of CREB binding protein (CBP) in response to activated Akt pathway [44], making CBP a likely cofactor that might potentiate PPARγ response to ghrelin-activated Akt pathway. Clearly, further studies are needed to identify the exact activation mechanism by which PPARγ responds to enhanced PI3-K/Akt pathway in macrophages, and how this process might selectively be facilitated through cofactor usage.

Collectively, our results establish a GHS-R1a signaling pathway that functions to regulate PPARγ transcriptional potential in macrophages, which implicates ghrelin and GHS-R1a in the removal of cholesterol from vascular atherosclerotic lesions. In line with such beneficial role of macrophage GHS-R1a receptor, we previously demonstrated the anti-atherogenic properties of hexarelin, a hexapeptide ligand that binds to both scavenger CD36 and GHS-R1a receptors [26], [58]. Given that ghrelin also decreases the release of proinflammatory cytokines in monocytes and T lymphocytes from T cells and monocytes [59], our findings raise the interesting possibility that ghrelin might have the potential to reduce atherosclerotic lesion development, which might represent an important mechanism for the recognized cardiovascular protection of ghrelin observed in animal models and humans [8]–[10]. In addition, it seems reasonable to speculate that the Dok-1 mediated inhibition of Erk1/2 could also predict the anti-atherogenic effects of ghrelin, given the high impact of elevated MAPK pathway in atherosclerosis [60], [61]. Altogether, our findings provide a cellular basis for the peripheral actions of ghrelin and the immediate response of PPARγ-regulated pathways that have potential important implications for the treatment of human vascular diseases.

## Methods

### Plasmids Constructs

pCMX expression plasmids coding for mouse PPARγ2, human PPARγ1, and Gal4- PPARγABCD (aa 1–254) have been described previously [26]. The N-terminal truncated Gal4-PPARγCDEF construct was generated by amplifying the aa 109-477 fragment of hPPARγ1 by PCR. The PPARγ1 Ser-84 to alanine and the PPARγ2 Ser-112 to alanine mutants were generated by PCR mutagenesis and confirmed by automated sequencing. The luciferase reporter constructs UAStkLuc and PPREtkLuc, as well as plasmids for Akt and its inactive K179M mutant, and for constitutively active p110α catalytic subunit of PI3-K have been described [26], [44]. Expression plasmids coding for Fyn kinase and Dok-1 were kindly provided by M. Resh and P. Duplay respectively.

### Cell Culture

Human monocytes THP-1 cells (ATCC, Manassas, VA) were grown in RPMI 1640 supplemented with 10% FBS. Macrophage differentiation was initiated by the addition of 5 ng/ml phorbol myristate acetate (PMA) in culture medium for 48–72 h. Human embryonic kidney 293 cells were cultured in DMEM containing 5% fetal bovine serum (FBS). Treatments with rosiglitazone (1–1.4 µM) and ghrelin (1–100 nM) were replaced with fresh medium every 24 h.

### RNA Isolation and qPCR Analysis

Total RNA was isolated from THP-1 cells using TRIzol reagent (Invitrogen, Burlington, Ontario, Canada) and RT-PCR analysis was done as described [62], [63]. PCR products were analyzed on a MX3000P (Stratagene, La Jolla, CA) and on gel (Alpha Innotech, San Leandro, CA), from at least three separate experiments. All values were normalized against GAPDH expression.

### RNA Interference

To silence PPARγ expression, small hairpin RNA duplexes targeting the sequence AAAGCAAAGGCGAGGGCGATCTT of human PPARγ (shPPARγ) were inserted into the pLVTH lentiviral vector for small interfering RNA production. Viral particles were produced in 293T cells as described [28], [63], and used to infect THP-1 macrophages. PPARγ efficient knockdown was monitored by Western analysis (data not shown).

### Antibodies

Antibodies to PPARγ, LXRα, Fyn, phospho-Akt (Ser-473), phospho-p85, p85, Dok-1, and phospho-tyrosine were obtained from Santa Cruz Biotechnology (Santa Cruz, CA). Antibodies to ABCG1 were obtained from Novus Biologicals (Littleton, CO), anti-PPARγ phospho-Ser-84 from Upstate (Lake Placid, NY), anti-Src phospho-Tyr-418 from Calbiochem (Darmstadt, Germany), anti-Erk1/2 from Invitrogen (Carlsbad, CA), and anti-β-actin from Abcam (Cambridge, MA). The anti-phospho-serine antibody was obtained from Chemicon (Temecula, CA), and the anti-Akt from Cell Signaling Technology (Danvers, MA). Antibodies against CD36 and GHS-R1a [26], [64], and HA [63], [65] have been described.

### Immunoprecipitation and Immunoblotting Analysis

Immunoprecipitation and immunoblotting procedures were done essentially as described [27], [66]. Briefly, for immunoprecipitation assay, cells were washed in ice-cold PBS, and lysed in RIPA buffer consisting of 1% Triton X-100, 0.5% deoxycholic acid, 0.1% sodium dodecyl sulfate (SDS), 1 mM sodium fluoride, 1 mM sodium orthovanadate, 1 mM phenylmethylsulfonyl fluoride, and protease inhibitors (Roche, Laval, Qc) in PBS. Cell lysates were precleared before incubation with respective antibodies and protein A-agarose at 4°C. Immunoprecipitates were then washed in lysis buffer, resolved by SDS-PAGE, and analyzed by immunoblotting. For coimmunoprecipitation assay, cells lysates were lysed in a modified RIPA containing 1% NP-40 and 0.25% deoxycholic acid. Proteins were then resolved by SDS-PAGE and subjected to Western analysis. Membranes were blocked with blocking reagent (Roche) or 5% nonfat dry milk in Tris-buffered saline, probed with selected antibodies, and signals visualized by enhanced chemiluminescence using appropriate horseradish peroxidase-conjugated secondary antibodies.

### Luciferase Reporter Assay

For transient transfection, 293 cells were seeded in DMEM supplemented with 5% charcoal dextran-treated FBS, and plasmid constructs were introduced into cells using the calcium phosphate precipitation method essentially as described [65]. Typically, 500 ng of reporter plasmid, 100 ng of nuclear receptor expression vector, 20–100 ng pcDNA-hGHS-R1a plasmid, and 200 ng of CMX-βgal were added per well. To activate the Akt pathway, cells were transfected with 100 ng each of PI3-K p110α and Akt expression vectors. After 6–8 h of transfection, cells were refed with medium containing 1–100 nM ghrelin or 1.4 µM rosiglitazone for 16–20 h or left untreated. For luciferase assay, cells were lysed in potassium phosphate buffer containing 1% Triton X-100, and light emission was measured using a luminometer (Wallac, Turku, Finland) after the addition of luciferin. Luciferase values were normalized for transfection efficiency to β-galactosidase activity of each sample, and expressed as relative fold response compared with controls. Luciferase assays were done in triplicates from at least three independent experiments.

## References

[1] Kojima M, Hosoda H, Date Y, Nakazato M, Matsuo H (1999). Ghrelin is a growth-hormone-releasing acylated peptide from stomach.. Nature.

[2] van der Lely AJ, Tschop M, Heiman ML, Ghigo E (2004). Biological, physiological, pathophysiological, and pharmacological aspects of ghrelin.. Endocr Rev.

[3] Howard AD, Feighner SD, Cully DF, Arena JP, Liberator PA (1996). A receptor in pituitary and hypothalamus that functions in growth hormone release.. Science.

[4] Chen C, Wu D, Clarke IJ (1996). Signal transduction systems employed by synthetic GH-releasing peptides in somatotrophs.. J Endocrinol.

[5] Katugampola SD, Pallikaros Z, Davenport AP (2001). [125I-His(9)]-ghrelin, a novel radioligand for localizing GHS orphan receptors in human and rat tissue: up-regulation of receptors with athersclerosis.. Br J Pharmacol.

[6] Gnanapavan S, Kola B, Bustin SA, Morris DG, McGee P (2002). The tissue distribution of the mRNA of ghrelin and subtypes of its receptor, GHS-R, in humans.. J Clin Endocrinol Metab.

[7] Baldanzi G, Filigheddu N, Cutrupi S, Catapano F, Bonissoni S (2002). Ghrelin and des-acyl ghrelin inhibit cell death in cardiomyocytes and endothelial cells through ERK1/2 and PI 3-kinase/AKT.. J Cell Biol.

[8] Garcia EA, Korbonits M (2006). Ghrelin and cardiovascular health.. Curr Opin Pharmacol.

[9] Shimizu Y, Nagaya N, Teranishi Y, Imazu M, Yamamoto H (2003). Ghrelin improves endothelial dysfunction through growth hormone-independent mechanisms in rats.. Biochem Biophys Res Commun.

[10] Chorny A, Anderson P, Gonzalez-Rey E, Delgado M (2008). Ghrelin protects against experimental sepsis by inhibiting high-mobility group box 1 release and by killing bacteria.. J Immunol.

[11] Iantorno M, Chen H, Kim JA, Tesauro M, Lauro D (2007). Ghrelin has novel vascular actions that mimic PI 3-kinase-dependent actions of insulin to stimulate production of NO from endothelial cells.. Am J Physiol Endocrinol Metab.

[12] Li WG, Gavrila D, Liu X, Wang L, Gunnlaugsson S (2004). Ghrelin inhibits proinflammatory responses and nuclear factor-kappaB activation in human endothelial cells.. Circulation.

[13] Li A, Cheng G, Zhu GH, Tarnawski AS (2007). Ghrelin stimulates angiogenesis in human microvascular endothelial cells: Implications beyond GH release.. Biochem Biophys Res Commun.

[14] Camina JP, Lodeiro M, Ischenko O, Martini AC, Casanueva FF (2007). Stimulation by ghrelin of p42/p44 mitogen-activated protein kinase through the GHS-R1a receptor: role of G-proteins and beta-arrestins.. J Cell Physiol.

[15] Rossi F, Castelli A, Bianco MJ, Bertone C, Brama M (2008). Ghrelin induces proliferation in human aortic endothelial cells via ERK1/2 and PI3K/Akt activation.. Peptides.

[16] Xu X, Jhun BS, Ha CH, Jin ZG (2008). Molecular mechanisms of ghrelin-mediated endothelial nitric oxide synthase activation.. Endocrinology.

[17] Filigheddu N, Gnocchi VF, Coscia M, Cappelli M, Porporato PE (2007). Ghrelin and des-acyl ghrelin promote differentiation and fusion of C2C12 skeletal muscle cells.. Mol Biol Cell.

[18] Rossi F, Castelli A, Bianco MJ, Bertone C, Brama M (2009). Ghrelin inhibits contraction and proliferation of human aortic smooth muscle cells by cAMP/PKA pathway activation.. Atherosclerosis.

[19] Chawla A, Boisvert WA, Lee CH, Laffitte BA, Barak Y (2001). A PPAR gamma-LXR-ABCA1 pathway in macrophages is involved in cholesterol efflux and atherogenesis.. Mol Cell.

[20] Ricote M, Valledor AF, Glass CK (2004). Decoding transcriptional programs regulated by PPARs and LXRs in the macrophage: effects on lipid homeostasis, inflammation, and atherosclerosis.. Arterioscler Thromb Vasc Biol.

[21] Castrillo A, Tontonoz P (2004). Nuclear Receptors in Macrophage Biology: At the Crossroads of Lipid Metabolism and Inflammation.. Annu Rev Cell Dev Biol.

[22] Li AC, Brown KK, Silvestre MJ, Willson TM, Palinski W (2000). Peroxisome proliferator-activated receptor gamma ligands inhibit development of atherosclerosis in LDL receptor-deficient mice.. J Clin Invest.

[23] Pasceri V, Wu HD, Willerson JT, Yeh ET (2000). Modulation of vascular inflammation in vitro and in vivo by peroxisome proliferator-activated receptor-gamma activators.. Circulation.

[24] Chen Z, Ishibashi S, Perrey S, Osuga J, Gotoda T (2001). Troglitazone inhibits atherosclerosis in apolipoprotein E-knockout mice: pleiotropic effects on CD36 expression and HDL.. Arterioscler Thromb Vasc Biol.

[25] Collins AR, Meehan WP, Kintscher U, Jackson S, Wakino S (2001). Troglitazone inhibits formation of early atherosclerotic lesions in diabetic and nondiabetic low density lipoprotein receptor-deficient mice.. Arterioscler Thromb Vasc Biol.

[26] Avallone R, Demers A, Rodrigue-Way A, Bujold K, Harb D (2006). A Growth Hormone-Releasing Peptide that Binds Scavenger Receptor CD36 and Ghrelin Receptor Upregulates ABC Sterol Transporters and Cholesterol Efflux in Macrophages Through a PPARγ-dependent Pathway.. Molecular Endocrinology.

[27] Marleau S, Harb D, Bujold K, Avallone R, Iken K (2005). EP80317, a ligand of the CD36 scavenger receptor, protects apolipoprotein E-deficient mice from developing atherosclerotic lesions.. FASEB J.

[28] Feige JN, Gelman L, Rossi D, Zoete V, Metivier R (2007). The endocrine disruptor mono-ethyl-hexyl-phthalate is a selective PPARgamma modulator which promotes adipogenesis.. J Biol Chem.

[29] Adams M, Reginato MJ, Shao D, Lazar MA, Chatterjee VK (1997). Transcriptional activation by peroxisome proliferator-activated receptor γ is inhibited by phosphorylation at a consensus mitogen- activated protein kinase site.. Journal of Biological Chemistry.

[30] Hu E, Kim JB, Sarraf P, Spiegelman BM (1996). Inhibition of adipogenesis through MAP kinase-mediated phosphorylation of PPARγ.. Science.

[31] Rozengurt E (2007). Mitogenic signaling pathways induced by G protein-coupled receptors.. J Cell Physiol.

[32] Nanzer AM, Khalaf S, Mozid AM, Fowkes RC, Patel MV (2004). Ghrelin exerts a proliferative effect on a rat pituitary somatotroph cell line via the mitogen-activated protein kinase pathway.. Eur J Endocrinol.

[33] Chung S, Lapoint K, Martinez K, Kennedy A, Boysen SM (2006). Preadipocytes mediate lipopolysaccharide-induced inflammation and insulin resistance in primary cultures of newly differentiated human adipocytes.. Endocrinology.

[34] McGarrigle D, Huang XY (2007). GPCRs signaling directly through Src-family kinases.. Sci STKE.

[35] Loppnow H, Werdan K, Buerke M (2008). Vascular cells contribute to atherosclerosis by cytokine- and innate-immunity-related inflammatory mechanisms.. Innate Immun.

[36] Reddy MA, Sahar S, Villeneuve LM, Lanting L, Natarajan R (2009). Role of Src tyrosine kinase in the atherogenic effects of the 12/15-lipoxygenase pathway in vascular smooth muscle cells.. Arterioscler Thromb Vasc Biol.

[37] Bastie CC, Zong H, Xu J, Busa B, Judex S (2007). Integrative metabolic regulation of peripheral tissue fatty acid oxidation by the SRC kinase family member Fyn.. Cell Metab.

[38] Shao D, Rangwala SM, Bailey ST, Krakow SL, Reginato MJ (1998). Interdomain communication regulating ligand binding by PPAR-γ.. Nature (London).

[39] Carpino N, Wisniewski D, Strife A, Marshak D, Kobayashi R (1997). p62(dok): a constitutively tyrosine-phosphorylated, GAP-associated protein in chronic myelogenous leukemia progenitor cells.. Cell.

[40] Yamanashi Y, Baltimore D (1997). Identification of the Abl- and rasGAP-associated 62 kDa protein as a docking protein, Dok.. Cell.

[41] Feuillet V, Semichon M, Restouin A, Harriague J, Janzen J (2002). The distinct capacity of Fyn and Lck to phosphorylate Sam68 in T cells is essentially governed by SH3/SH2-catalytic domain linker interactions.. Oncogene.

[42] Shinohara H, Yasuda T, Yamanashi Y (2004). Dok-1 tyrosine residues at 336 and 340 are essential for the negative regulation of Ras-Erk signalling, but dispensable for rasGAP-binding.. Genes Cells.

[43] Boulay I, Nemorin JG, Duplay P (2005). Phosphotyrosine binding-mediated oligomerization of downstream of tyrosine kinase (Dok)-1 and Dok-2 is involved in CD2-induced Dok phosphorylation.. J Immunol.

[44] Sanchez M, Sauvé K, Picard N, Tremblay A (2007). The hormonal response of estrogen receptor beta is decreased by the PI3K/Akt pathway via a phosphorylation-dependent release of CREB-binding protein.. J Biol Chem.

[45] Falls HD, Dayton BD, Fry DG, Ogiela CA, Schaefer VG (2006). Characterization of ghrelin receptor activity in a rat pituitary cell line RC-4B/C.. J Mol Endocrinol.

[46] Wettschureck N, Moers A, Wallenwein B, Parlow AF, Maser-Gluth C (2005). Loss of Gq/11 family G proteins in the nervous system causes pituitary somatotroph hypoplasia and dwarfism in mice.. Mol Cell Biol.

[47] Tesauro M, Schinzari F, Iantorno M, Rizza S, Melina D (2005). Ghrelin improves endothelial function in patients with metabolic syndrome.. Circulation.

[48] Camp HS, Tafuri SR (1997). Regulation of peroxisome proliferator-activated receptor γ activity by mitogen-activated protein kinase.. Journal of Biological Chemistry.

[49] Zhang B, Berger J, Zhou G, Elbrecht A, Biswas S (1996). Insulin- and mitogen-activated protein kinase-mediated phosphorylation and activation of peroxisome proliferator-activated receptor gamma.. J Biol Chem.

[50] Rangwala SM, Rhoades B, Shapiro JS, Rich AS, Kim JK (2003). Genetic modulation of PPARgamma phosphorylation regulates insulin sensitivity.. Dev Cell.

[51] Sun Y, Ma YC, Huang J, Chen KY, McGarrigle DK (2005). Requirement of SRC-family tyrosine kinases in fat accumulation.. Biochemistry.

[52] Hosooka T, Noguchi T, Kotani K, Nakamura T, Sakaue H (2008). Dok1 mediates high-fat diet-induced adipocyte hypertrophy and obesity through modulation of PPAR-gamma phosphorylation.. Nat Med.

[53] Barazzoni R, Bosutti A, Stebel M, Cattin MR, Roder E (2005). Ghrelin regulates mitochondrial-lipid metabolism gene expression and tissue fat distribution in liver and skeletal muscle.. Am J Physiol Endocrinol Metab.

[54] Lodeiro M, Theodoropoulou M, Pardo M, Casanueva FF, Camina JP (2009). c-Src regulates Akt signaling in response to ghrelin via beta-arrestin signaling-independent and -dependent mechanisms.. PLoS ONE.

[55] Magun R, Burgering BM, Coffer PJ, Pardasani D, Lin Y (1996). Expression of a constitutively activated form of protein kinase B (c-Akt) in 3T3-L1 preadipose cells causes spontaneous differentiation.. Endocrinology.

[56] Uto-Kondo H, Ohmori R, Kiyose C, Kishimoto Y, Saito H (2009). Tocotrienol suppresses adipocyte differentiation and Akt phosphorylation in 3T3-L1 preadipocytes.. J Nutr.

[57] Feige JN, Gelman L, Michalik L, Desvergne B, Wahli W (2006). From molecular action to physiological outputs: Peroxisome proliferator-activated receptors are nuclear receptors at the crossroads of key cellular functions.. Prog Lipid Res.

[58] Demers A, Rodrigue-Way A, Tremblay A (2008). Hexarelin Signaling to PPARgamma in Metabolic Diseases.. PPAR Res.

[59] Dixit VD, Yang H, Cooper-Jenkins A, Giri BB, Patel K (2009). Reduction of T cell-derived ghrelin enhances proinflammatory cytokine expression: implications for age-associated increases in inflammation.. Blood.

[60] Rahaman SO, Lennon DJ, Febbraio M, Podrez EA, Hazen SL (2006). A CD36-dependent signaling cascade is necessary for macrophage foam cell formation.. Cell Metab.

[61] Muslin AJ (2008). MAPK signalling in cardiovascular health and disease: molecular mechanisms and therapeutic targets.. Clin Sci (Lond).

[62] Rodrigue-Way A, Demers A, Ong H, Tremblay A (2007). A Growth Hormone-Releasing Peptide Promotes Mitochondrial Biogenesis And A Fat Burning-like Phenotype Through Scavenger Receptor CD36 in White Adipocytes.. Endocrinology.

[63] Sauvé K, Lepage J, Sanchez M, Heveker N, Tremblay A (2009). Positive Feedback Activation of Estrogen Receptors by the CXCL12-CXCR4 Pathway.. Cancer Res.

[64] Demers A, McNicoll N, Febbraio M, Servant M, Marleau S (2004). Identification of the growth hormone-releasing peptide binding site in CD36: a photoaffinity cross-linking study.. Biochem J.

[65] Picard N, Charbonneau C, Sanchez M, Licznar A, Busson M (2008). Phosphorylation of activation function-1 regulates proteasome-dependent nuclear mobility and E6-AP ubiquitin ligase recruitment to the estrogen receptor beta.. Mol Endocrinol.

[66] St Laurent V, Sanchez M, Charbonneau C, Tremblay A (2005). Selective hormone-dependent repression of estrogen receptor beta by a p38-activated ErbB2/ErbB3 pathway.. J Steroid Biochem Mol Biol.

